# Exceptional Manifestation of Multiple Myeloma

**DOI:** 10.7759/cureus.42881

**Published:** 2023-08-03

**Authors:** Afaf Thouil, Meriem Rhazari, Sara Gartini, Hatim Kouismi

**Affiliations:** 1 Department of Respiratory Diseases, Research and Medical Sciences Laboratory, Faculty of Medicine and Pharmacy of Oujda, Mohammed VI University Hospital, Mohammed First University, Oujda, MAR; 2 Department of Pulmonology, Mohammed VI University Hospital, Oujda, MAR

**Keywords:** kappa band, myelogram, pleural immunofixation, pleurisy, multiple myeloma

## Abstract

Despite being a rare occurrence, multiple myeloma (MM) has been reported as an alternative cause of pleurisy, with approximately 50 documented cases in the literature so far. In this case report, we present the clinical scenario of a patient who sought medical attention due to symptoms of dyspnea, chest pain, and weight loss. Through a comprehensive diagnostic evaluation, it was determined that the patient's pleural involvement was attributable to MM, a hematological malignancy. This case highlights the importance of considering MM as a potential etiology in patients presenting with pleural manifestations, even in settings where tuberculosis is the prevailing cause.

## Introduction

Multiple myeloma (MM) is a malignant neoplasm of the hematopoietic system, characterized by the uncontrolled proliferation of plasma cells that infiltrate the bone marrow, leading to the disruption of normal hematopoiesis. This hematological malignancy is marked by the clonal expansion of abnormal plasma cells, which produce excessive amounts of monoclonal immunoglobulins. Clinically, MM presents with a spectrum of manifestations, with the most prevalent being anemia, hypercalcemia, renal insufficiency, and skeletal fractures [1]. These clinical features result from the direct effects of the abnormal plasma cells on the bone marrow, as well as the systemic consequences of monoclonal protein production and its associated complications. It has been observed that MM primarily affects elderly individuals, and many studies highlight that the incidence of MM increases with advancing age, with the majority of cases occurring in patients over the age of 65 [2,3]. Pleurisy in MM is a relatively uncommon occurrence, with an estimated frequency of 6%. Its presence is often associated with a poor prognosis, indicating advanced disease and a potentially more aggressive clinical course. While pleurisy in MM is rarely of plasma cell origin, it can be exceptionally revelatory, serving as a significant diagnostic clue to the underlying malignancy in some cases. The most common causes of pleurisy in MM are amyloidosis and heart failure, while pulmonary infarctions are uncommon in this context. Understanding the clinical manifestations and potential causes of pleurisy in MM is essential for appropriate management and prognostic assessment of the affected patients [4].

## Case presentation

**Figure 1:**
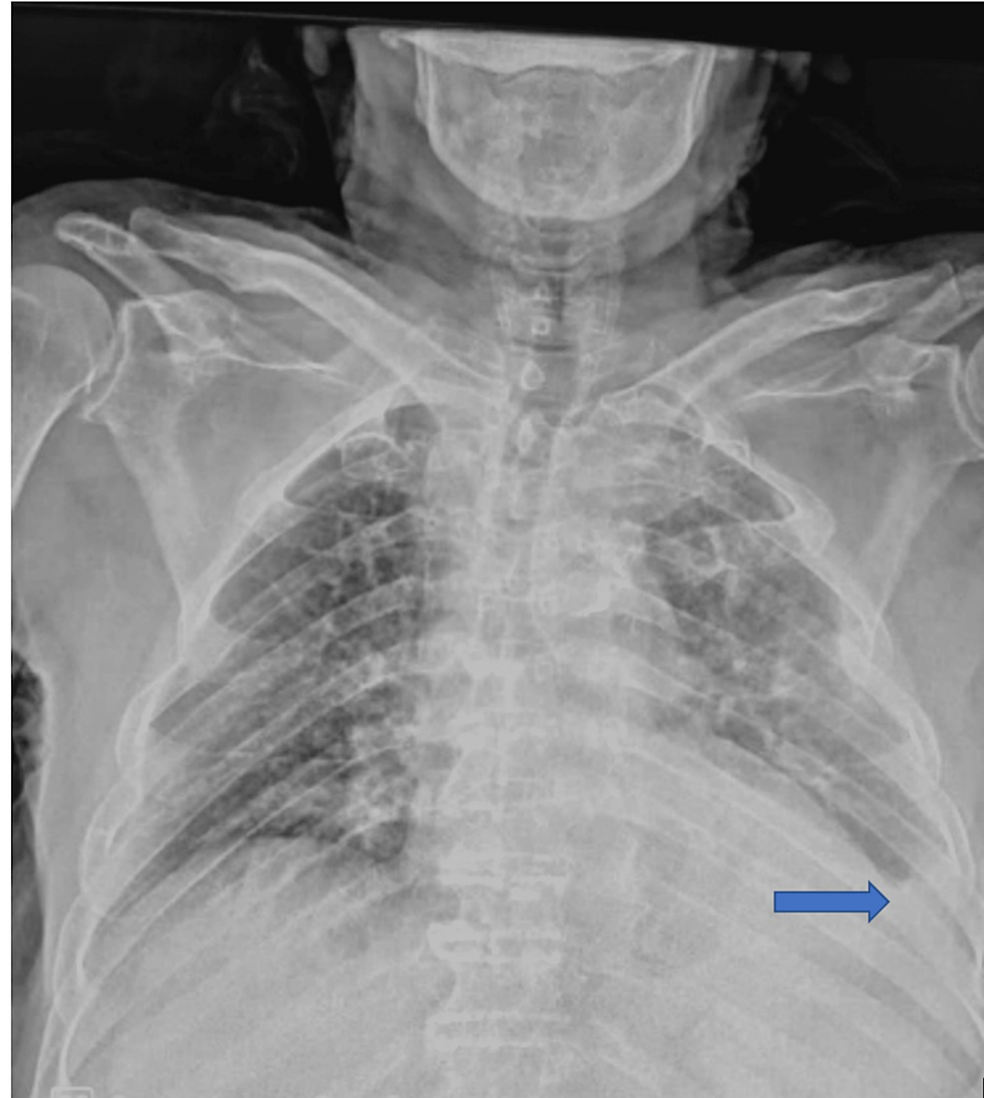
Chest X-ray showing a low-volume left-sided pleural effusion (blue arrow).

We present a noteworthy case of MM that was initially manifested as pleurisy, that is, the case of a 95-year-old male with a history of chronic smoking, insulin-dependent diabetes mellitus, and hypothyroidism managed with levothyroxine. The patient experienced left-sided chest pain and gradually worsening exertional dyspnea over the past two months. Upon physical examination, the patient appeared to be hemodynamically stable and respiratory-wise, with arterial oxygen saturation (AA) at 90% and the presence of left basal pleural fluid effusion syndrome. Chest X-ray (Figure 1) showed an image of low-volume left pleurisy.

The hemogram revealed normocytic normochromic anemia (Hb = 9.3 g/dL) with rouleaux formation of red blood cells. The sedimentation rate (SR) was elevated at 140 mm. The corrected blood calcium level was 95 mg/L and the renal function was normal. Pleural puncture yielded a dark citrine exudative fluid with a protein level of 83.2 g/L. At this point we considered different etiologies of exudative pleuritis, and among them was MM. The pleural biopsy revealed fibro-inflammatory remodeling.

**Figure 2:**
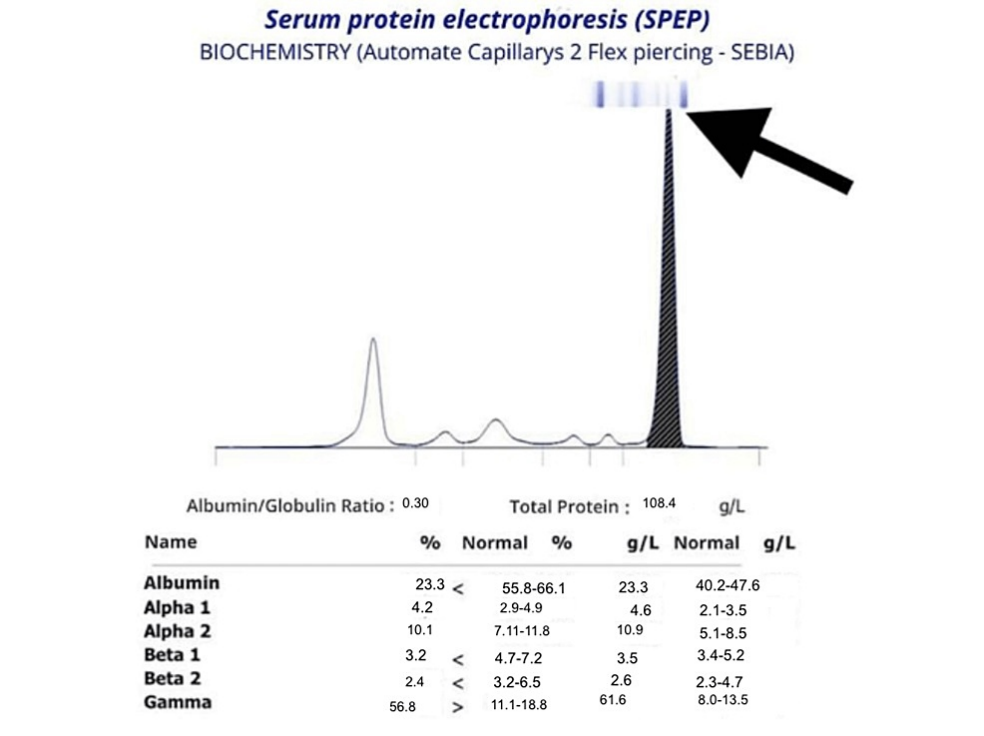
The electrophoretic profile showed a peak (arrow) quantified at 100 g/L, suspiciously migrating in the gamma globulin region. Hypoalbuminemia and hyperalpha-2 globulinemia were also observed.

Given the association of anemia with rouleaux formation of red blood cells, elevated SR, and costal lysis, the diagnosis of MM was suspected. This led to serum protein electrophoresis (Figure 2), which revealed a peak quantified at 100 g/L, suspiciously migrating into the gamma globulin area. Hypoalbuminemia and hyper alpha-2 globulinemia were also observed.

**Figure 3:**
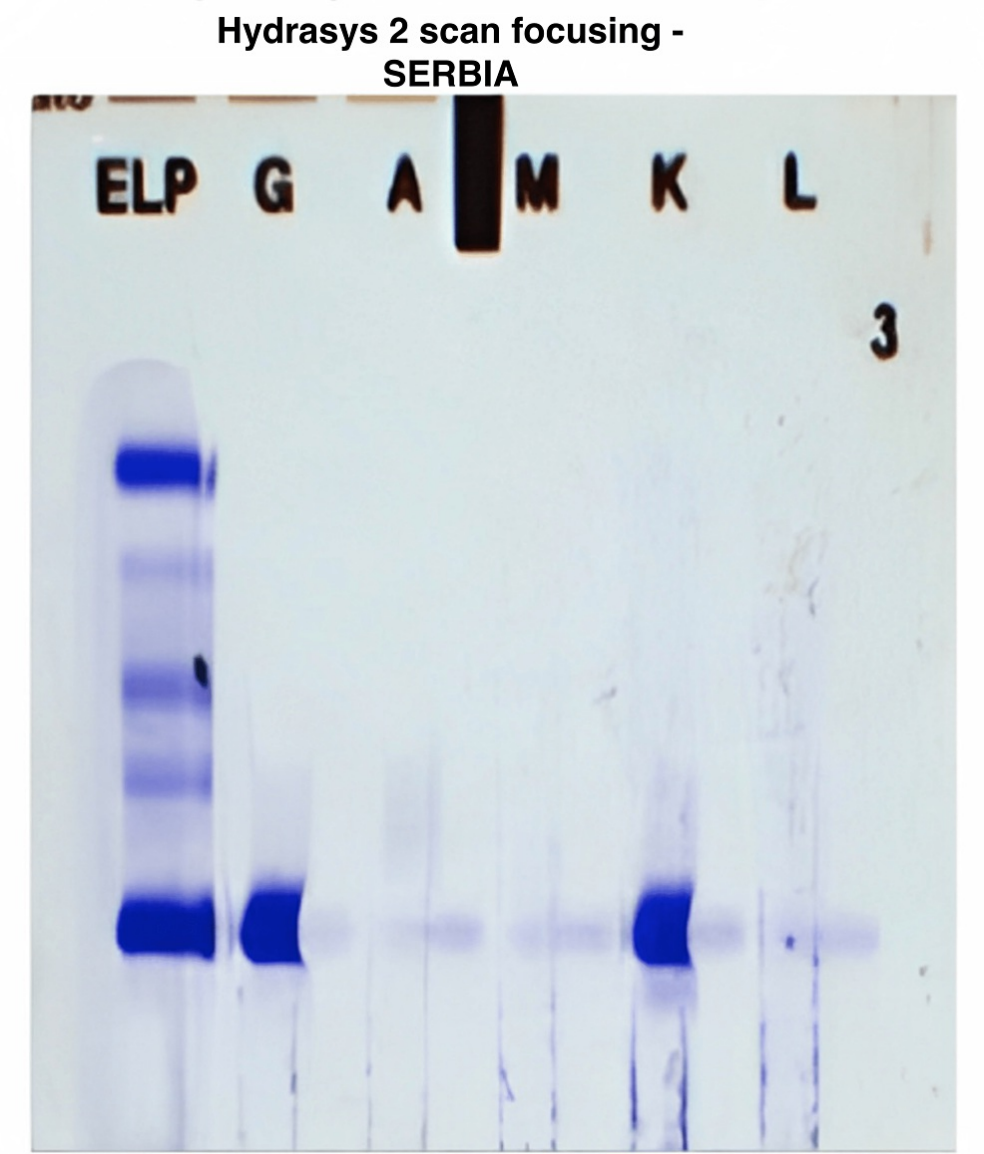
Serum immunofixation showing an IgG kappa band

**Figure 4:**
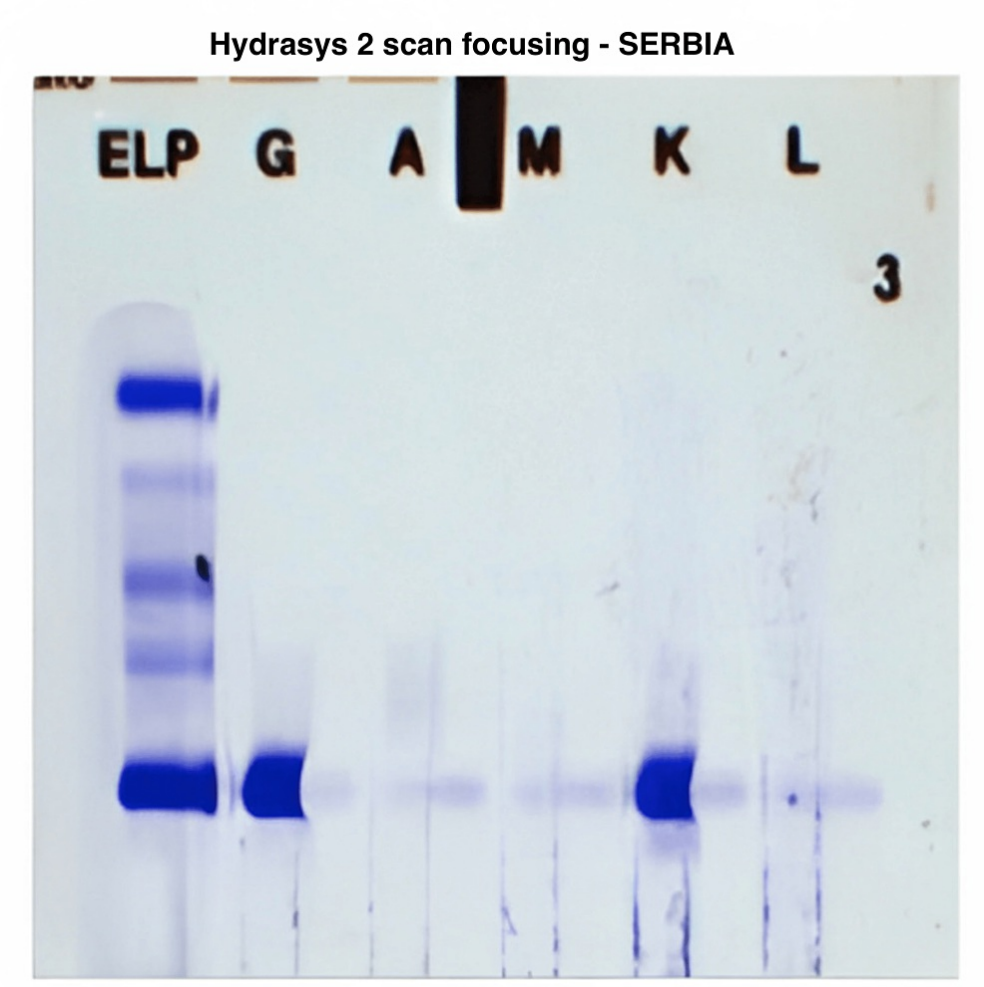
Urine immunofixation showing a kappa band with a free kappa band with a match at the G.A.M.

**Figure 5:**
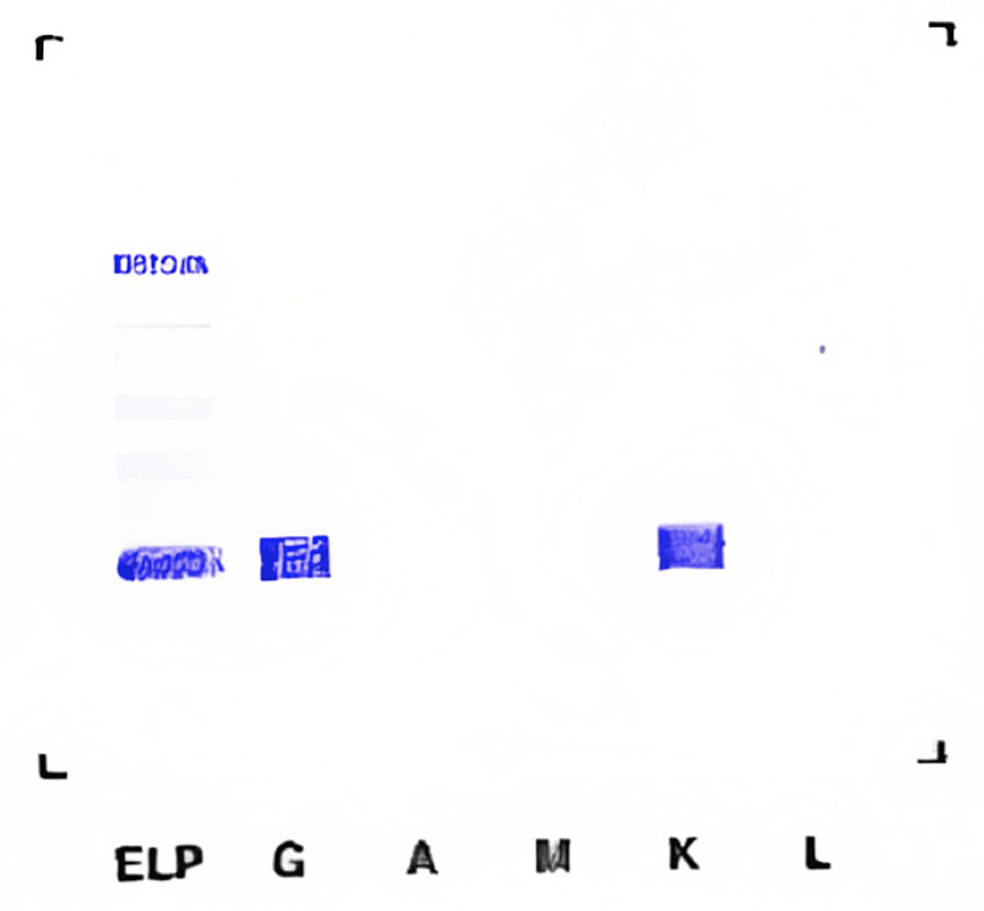
Pleural immunofixation: the presence of monoclonal immunoglobulin of the IgG kappa type

Serum, urine, and pleural immunoelectrophoresis (Figures 3-5) revealed the presence of IgG kappa immunoglobulin.

**Figure 6:**
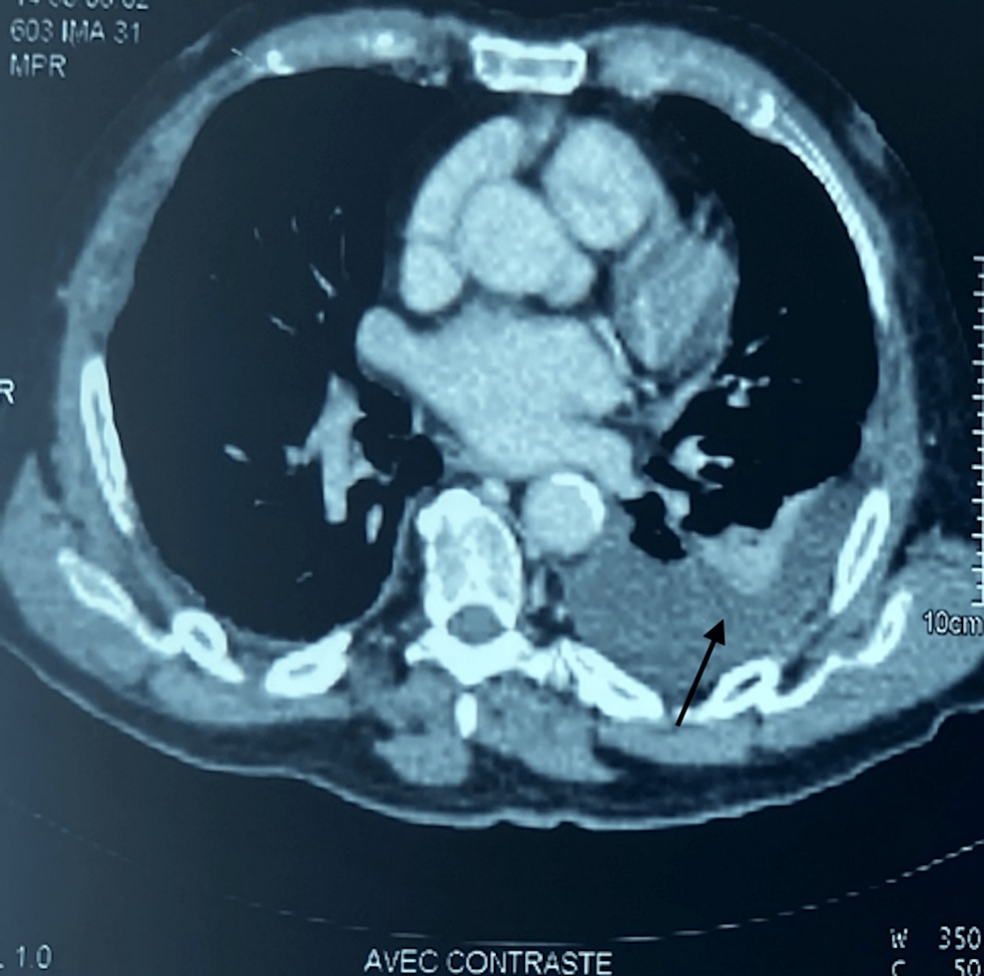
The chest CT scan revealed a collapsed lung along with a moderate-sized pleural fluid effusion (arrow) on the left side

The thoracic CT scan (Figure 6) reveals a lung collapse accompanied by a pleural effusion and evidence of sternum and left third costal arch lysis.

Based on the findings from the previous examinations, additional investigations were conducted, including skeletal X-ray, electrocardiogram, cardiac ultrasound, myelogram, and bone marrow biopsy. The skeletal X-ray and electrocardiogram showed normal results, while the cardiac ultrasound revealed no abnormalities. The myelogram indicated a bone marrow rich in plasma cells, and the bone marrow biopsy confirmed histological and immunohistochemical evidence of marrow infiltration, characterized by plasma cell proliferation consistent with a diagnosis of MM.

Taking into consideration that Morocco is a country with tuberculosis endemicity, tuberculosis infection was initially considered due to the patient's presentation. However, after ruling out tuberculosis and conducting all the mentioned examinations in the patient's history, the diagnosis of MM was established based on the diagnostic criteria [5].

The final diagnosis was IgG kappa myeloma, with multiple bone, urinary, and pleural involvements, classified as stage III according to the Durie and Salmon classification [5]. The patient was referred to internal medicine for appropriate management and care. Unfortunately, the patient passed away before initiating chemotherapy.

## Discussion

MM, also known as Kahler's disease, is a hematological malignancy characterized by the proliferation of a clone of tumor plasma cells invading the bone marrow and producing monoclonal immunoglobulin [6-8]. Bone manifestations are prominent in this disease. The diagnosis of MM is based on the presence of bone marrow plasma cell disease, significant levels of monoclonal immunoglobulin in the serum and/or urine, and clinical signs related to malignant plasma cell proliferation. Pleurisy is a rare manifestation in MM, occurring in approximately 6% of cases [2,6,9]. Furthermore, pleurisy is rarely indicative of myeloma [6,10], as observed in our case.

In MM, pleurisy can have various etiologies, with heart failure secondary to amyloid myocardial infiltration being the predominant cause [9]. Less frequently, pulmonary embolism, infection, and renal failure may contribute to pleurisy [2,6]. Plasma cell infiltration of the pleura is rare, occurring in only 1% of myeloma cases [2,6,9], and there have been approximately 50 published cases so far [6,11]. One of the reported cases was that of a 67-year-old woman with stage IIa IgA λ-type MM who was admitted to the hospital due to worsening dyspnea over one month [6].

A diagnostic pleural puncture is crucial and typically reveals the presence of monoclonal immunoglobulin of the same type as in the blood, along with abnormal plasma cells. This confirms the myeloma-related etiology of pleurisy while excluding other potential causes. The diagnosis is usually based on two main criteria [1,12]: the demonstration of identical monoclonal immunoglobulin in both the blood and pleural fluid, and the presence of abnormal plasma cells in the pleural effusion. In our case, the pleural effusion was indicative of myeloma, and the presence of the same type of monoclonal immunoglobulin was confirmed in the blood, urine, and pleural fluid, despite the absence of abnormal plasma cells.

The presence of plasma cell pleurisy and bone amyloidosis is associated with a poor prognosis [6,12]. The treatment of myeloma-related pleural effusions and bone amyloidosis lacks standardization and typically involves systemic chemotherapy with corticosteroids and therapeutic drainage procedures. Several treatment protocols have been attempted, such as melphalan-prednisone or melphalan-vincristine, cyclophosphamide-prednisone, or vincristine plus adriamycin-dexamethasone, but their efficacy has often been limited [9,10,12]. Some cases have reported regression of myeloma effusion following chemotherapy or radiotherapy [12]. Local treatments involving intrapleural injections of corticosteroids, adriamycin, or interferon have been attempted but without significant results [10,12]. Talc pleurodesis is performed in cases of recurrent pleurisy, providing temporary stabilization [9,11,12].

Unfortunately, our patient passed away before chemotherapy could be initiated. The prognosis of myeloma depends on the tumor burden and clinical stage. Durie and Salmon proposed a classification system based on tumor mass and prognosis. Treated patients have a median survival of over five years in stage I, 20 months in stage III, and intermediate survival in stage II. Other prognostic factors include the level of beta-2-microglobulin (considered unfavorable when exceeding 3 mg/L) and the type of immunoglobulin (increasing severity: IgG (+), IgA (++), BJ (+++), IgD (++++)). These factors have proven to be the most useful in predicting prognosis. The presence of plasma pleurisy further worsens the prognosis [2,6,9], with an average survival of no more than four months from the time of plasma pleurisy detection [6,10]. Prolonged survival of up to 30 months has been reported [6,9].

## Conclusions

The goal of this case study was to show that it could be interesting to think about the association of myeloma and pleurisy in the case of a pleural effusion without a common explanatory context. Thus, after ruling out the infectious component, particularly tuberculosis, in our context, additional biological examinations such as immunofixation of serum proteins and fluid should be requested. 
